# Supplementary material for: Advanced molecular surveillance approaches for characterization of blood borne hepatitis viruses
Source: PLoS One. 2020 Jul 17;15(7):e0236046. doi: 10.1371/journal.pone.0236046 (PMC7367454; doi:10.1371/journal.pone.0236046)
Supplement: S1 Appendix — (PDF) [file pone.0236046.s001.pdf]

S1 Appendix

Materials & Methods

Pan-viral enrichment

SSRTIII-Nextera mNGS libraries from the HBV diversity panel were pooled and dried down in a vacuum centrifuge at low heat. Libraries were resuspended in 7 µl nuclease-free water, followed by the addition of 5 µl Blocker solution (Cot-1 DNA) and 1 µl of Universal Blockers and heating at 60°C for 10 minutes. During the incubation, a 28 µl probe hybridization mix was assembled, consisting of 20 µl Hybridization buffer heated at 65°C for 10 minutes and cooled to 25°C for 5 minutes, followed by the addition of 4 µl Pan-Viral Probes and 4 µl water. The 28 µl probe mix was warmed to 32°C for 2 min and denatured at 95°C for 2 min and snapped chilled for 5 min. The library pool was also warmed to 32°C for 3 min, denatured at 95°C for 5 min, and cooled to 25°C before combining with the Hybridization mix. Hybridization Enhancer (30 µl) was overlaid on top of the 40 µl reaction and brought to 70°C for ≥16 hour hybridization. The entire reaction (70 µl) was then rapidly transferred to 100 µl of streptavidin beads pre-equilibrated in Binding Buffer. Beads were kept in solution by gentle rotation for 30 minutes, then placed on a magnetic stand for 1 minute to remove unbound DNA. A total of three washes were performed (1 at 25°C and 2 at 48°C) followed by resuspension of beads in 21 µl of water and transfer to ice. The DNA-bound beads were then added to 25 µl of 2X KAPA HiFi HotStart ReadyMix and 5 µl of 10 µM Illumina Primer Mix. Captured viral sequences were amplified on a Perkin-Elmer 9700 Thermal Cycler under the following conditions: 1 cycle (98°C_0:45); 15 cycles (98°C_0:15, 60°C_0:30, 72°C_0:30); 1 cycle (72°C_1:00); hold at 4°C. PCR products were cleaned up with DNA purification beads and washed with 80% ethanol, followed by elution in 22 µl of water. PCR amplification was repeated as above using 20 µl of eluate (no SA beads) as template and purified once again.
