# Supplementary figures and images for: Advanced molecular surveillance approaches for characterization of blood borne hepatitis viruses
Source: PLoS One. 2020 Jul 17;15(7):e0236046. doi: 10.1371/journal.pone.0236046 (PMC7367454; doi:10.1371/journal.pone.0236046)

## Positive control log 4 cp/ml: Genome coverage

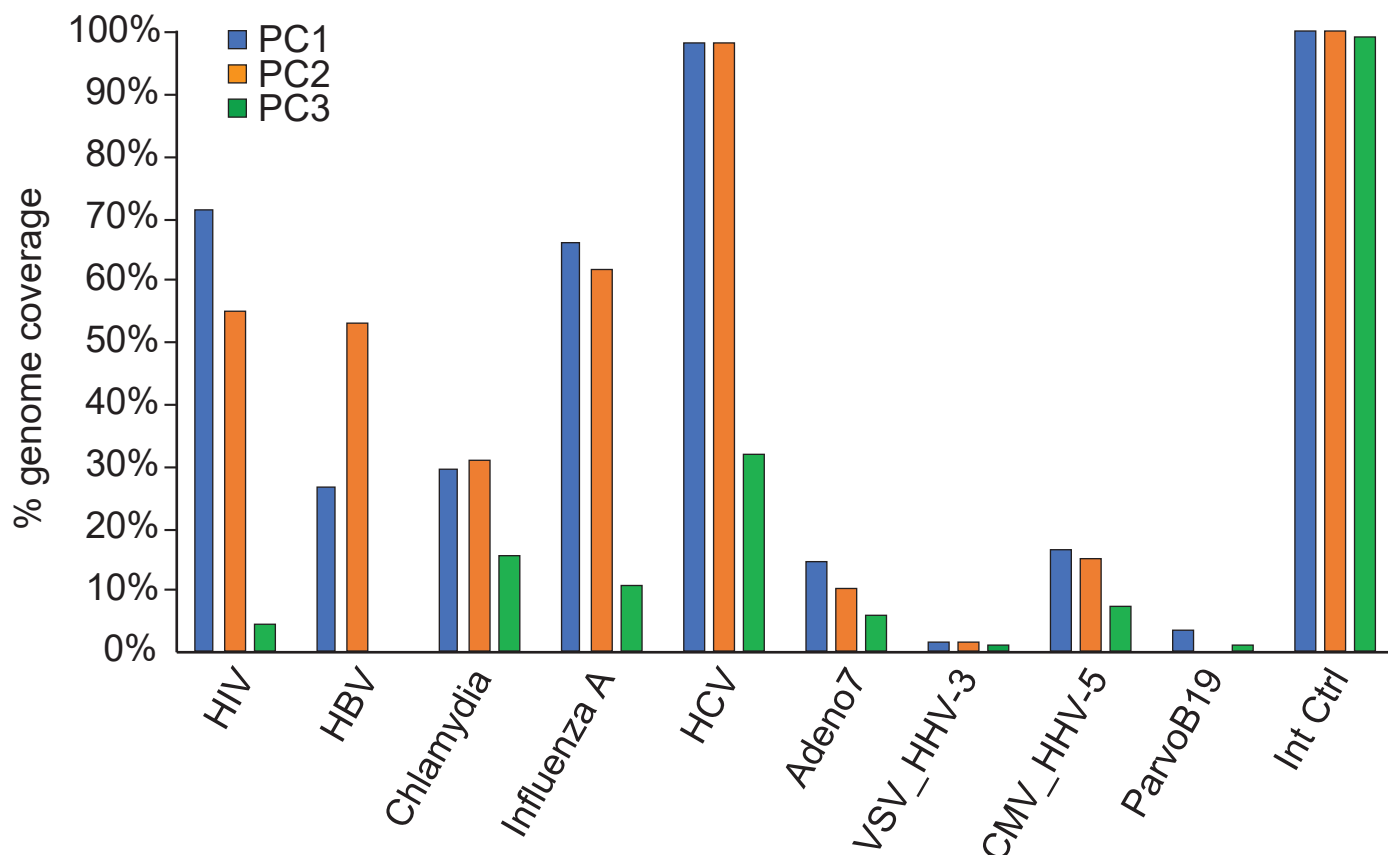

## Positive control log 4 cp/ml: Reads/million

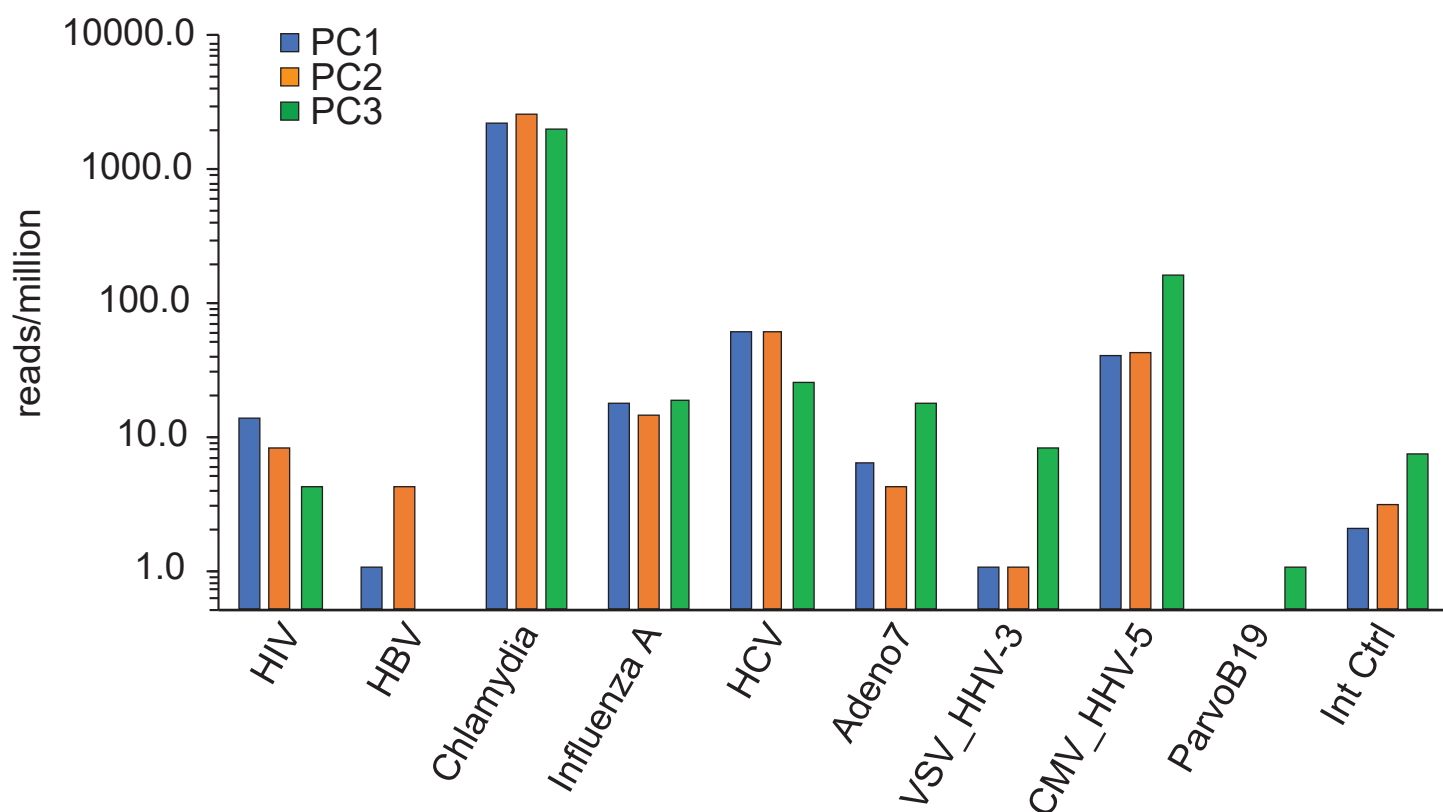

Supplement: S1 Fig — A positive control consisting of 8 viruses and chlamydia trachomatis spiked into normal human plasma each at log 4.0 copies/ml was included with samples in three separate extractions, library preps and sequencing runs of HCV positive samples. Reads were taxonomically assigned by the SURPI pipeline. The top histogram represents the genome coverages and the bottom histograms represent reads per million for each pathogen. (PDF) [file pone.0236046.s002.pdf]
