# Supplementary material for: Advanced molecular surveillance approaches for characterization of blood borne hepatitis viruses
Source: PLoS One. 2020 Jul 17;15(7):e0236046. doi: 10.1371/journal.pone.0236046 (PMC7367454; doi:10.1371/journal.pone.0236046)
Supplement: S1 Table — Demographic information for HCV and HBV/HDV positive patients enrolled in study at the Israeli National HIV and Viral Hepatitis Reference Center (NHRL). (PDF) [file pone.0236046.s003.pdf]

Sample_ID	Patient_ID	Virus	Age	Sex	Country of Birth	VL_HCV	Risk group
2000427	242844	HCV	82	F	Morocco	1291888	organ transplant
2000444	242924	HCV	56	M	FSU	n.d.	organ transplant
2000445	240370	HCV	38	F	Ukraine	23644	HETERO
2000446	242925	HCV	52	M	Latvia	1200000	Unknown
2000447	242942	HCV	62		Romenia	n.d.	blood transfusion
2000449	242948	HCV	80	M	Israel	1231418	Blood-hemophilia
2000463	243036	HCV	75	M	FSU	270520	Unknown
2000478	243091	HCV	56	M	FSU	n.d.	blood transfusion
2000484	243137	HCV	56	F	FSU	988359	Unknown
2000485	243141	HCV	81	F	Israel	n.d.	blood transfusion
2000487	243170	HCV	73	F	Israel	202000	Unknown
2000502	243242	HCV	70	M	Iraq	n.d.	Unknown
2000512	243322	HCV	63	M	Israel	878516	Unknown
2000513	243341	HCV	41	F	FSU	2258252	blood transfusion
2000516	243353	HCV	54	M	Morocco	n.d.	blood transfusion
2000517	243364	HCV	46	F	FSU	661302	Unknown
2000545	243717	HCV	70	F	FSU	291730	Unknown
2000554	243848	HCV	63	F	Romenia	1225671	blood transfusion
2000561	243910	HCV	63	F	Romenia	506624	organ transplant
2000572	244001	HCV	69	M	Israel	n.d.	IDU
2000573	244003	HCV	80	F	FSU	562026	Unknown
2000599	244235	HCV	69	F	Egypt	n.d.	blood transfusion
2000602	244240	HCV	78	F	Poland	n.d.	Unknown
2000607	244268	HCV	51	M	Israel	n.d.	Unknown
2000613	244310	HCV	58	M	FSU	121082	Unknown
2000615	244344	HCV	64	F	Moldova	1866050	Unknown
2000616	244342	HCV	36	M	Israel	n.d.	Unknown
2000618	244347	HCV	65	F	Georgia	n.d.	Unknown
2000619	244348	HCV	29	M	FSU	548348	Unknown
2000621	244351	HCV	50	F	Ukrain	1706864	Unknown
2000633	244431	HCV	54	F	FSU	142990	Unknown
2000637	244462	HCV	86	F	FSU	1529353	Unknown
2000646	244543	HCV	70	M	Iraq	607035	blood transfusion
2000650	244588	HCV	62	M	FSU	2559464	Unknown
2000668	244720	HCV	74	F	FSU	6016394	blood transfusion
2000669	244728	HCV	74	M	Romenia	884442	IDU
2000670	244740	HCV	54	M	Uzbekistan	498229	Unknown
2000672	244752	HCV	67	F	Morocco	60070015	Unknown
2000675	244769	HCV	70	M	Bulgaria	15769	blood transfusion
2000676	244788	HCV	64	F	Romenia	238486	blood transfusion
2000684	244934	HCV	53	M	FSU	3809424	Unknown
2000691	245007	HCV	19	M	Georgia	2460000	Unknown
2000692	245000	HCV	49	M	Ukraine	637000	Health worker
2000693	244998	HCV	61	F	FSU	1410000	blood transfusion
2000694	244996	HCV	50	F	FSU	1490000	Unknown
2000697	245017	HCV	45	M	unknown	n.d.	Unknown
2000705	245158	HCV	79	F	Russia	9339704	Unknown
2000726	245342	HCV	78	F	Ukraine	257436	blood transfusion
2000734	243037	HCV	71	F	FSU	4330156	Unknown
2000775	245614	HCV	76	M	FSU	4751713	blood transfusion
2000781	245661	HCV	39	F	Israel	12730	Unknown
2000782	245662	HCV	80	M	Ukraine	n.d.	Unknown
2000785	245669	HCV	63	F	Moldova	83277	Unknown
2000790	245698	HCV	75	M	Moldova	768300	blood transfusion
2000791	245708	HCV	54	F	FSU	n.d.	Unknown
2000798	245767	HCV	58	F	FSU	n.d.	Unknown
2000810	245853	HCV	52	M	Israel	n.d.	IDU
2000811	245851	HCV	70	M	Israel	11360455	Unknown
2000883	246370	HCV	31	M	France	n.d.	Unknown
2000887	246449	HCV	47	F	Romenia	n.d.	blood transfusion
2000889	246497	HCV	74	M	FSU	615416	Unknown
2000912	246885	HCV	53	M	Israel	19556	IDU
2000913	246911	HCV	70		Poland	459910	Unknown
2000929	247112	HCV	57	M	Morocco	2198680	Unknown
2000973	247507	HCV	56	F	Israel	3103008	blood transfusion
2000976	216892	HCV	44	M	Israel	452508	Blood-hemophilia
2000982	247632	HCV	44	F	FSU	488765	blood transfusion
2000984	247660	HCV	78	M	Iran	2502079	blood transfusion
2000986	247705	HCV	58	F	Ukraine	3.3.10^6	Unknown
2000989	247700	HCV	33	M	Ukraine	1931756	IDU
2001001	247768	HCV	68	F	Georgia	n.d.	blood transfusion
2001005	232803	HCV	31	F	unknown	n.d.	Unknown
2001011	247894	HCV	74	M	USA	n.d.	blood transfusion
2001044	248055	HCV	39		Israel	381340	Unknown
2001049	236209	HCV	31	M	FSU	7505522	IDU
2001052	217766	HCV	45	M	Israel	780010	Blood-hemophilia
2001065	248192	HCV	0	M	FSU	3239143	Health worker
2001067	248219	HCV	67	M	FSU	221114	blood transfusion
2001069	248224	HCV	62	F	FSU	252125	blood transfusion
2001070	215645	HCV	51	M	Israel	6144255	Blood-hemophilia
2001072	235194	HCV	38	M	Russia	13213911	IDU
2001079	248263	HCV	67	F	USA	512313	blood transfusion
2001088	248315	HCV	52	M	Ukraine	1753181	Unknown
2001092	248330	HCV	51	M	Ukraine	2294028	IDU
2001106	248398	HCV	49	M	Ukraine	1009648	Unknown
2001108	248400	HCV	63	F	Romenia	552945	blood transfusion
2001114	248417	HCV	81	F	Moldova	736509	blood transfusion
2001121	248423	HCV	60	F	Ukraine	237366	blood transfusion
2001123	240368	HCV	33	M	Israel	45579	MSM
2001129	248467	HCV	34	M	Russia	1430194	blood transfusion
2001133	248505	HCV	46	M	Israel	2.3x10^7	IDU
2001137	248555	HCV	59	M	Israel	1339334	IDU
2001144	248570	HCV	61	M	Israel	5290137	blood transfusion
2001159	248644	HCV	54	F	FSU	1580109	blood transfusion
2001160	248646	HCV	59	M	Israel	715743	IDU
2001172	225434	HCV	52	M	Israel	393937	Blood-hemophilia
2001206	229328	HCV	36	M	Germany	n.d.	MSM
2001218	248956	HCV	0	F	FSU	247641	Health worker
2001220	234810	HCV	50	M	Uzbekistan	184194	IDU

Sample_ID	Patient_ID	age	sex	Birth country	HBV VL (cps/m	HBV Ct	HDV VL/ ct	HDV Ct
2000166	243525	0	female	no info			46	
2000208	243553	31	female	Uzbekistan	148.45	34.64	1150000	
2000234	241470	50	male	Ukraine			248000	
2000236	243524	59	male	Russia	5.09	37.11	2140000	
2000319	243566	35	female	no info	0.99	42.94	54750	
2000320	243567	40	female	Russia	indeterminate		173563	
2000322	243568	55	female	no info	410.94	33.07	40000	
2000324	243569	34	female	Russia			43500	
2000372	243558	37	male	Russia			21800000	
2000568	243568	56	female	no info			1.3x10^6	
2000570	243983	41	female	Russia			1.24x10^9	
2000741	245513	50	female	Russia			349	
2000742	245514	46	male	Ethiopia	218.75	35.00	4.48x10^4	
2000744	245522	47	male	Russia	984.64	32.80	8.74x10^4	
2000999	243521	41	male	Russia			1.15x10^6	
2001063	243562	43	male	Russia			3.03x10^7	16.6
2001073	244398	39	male	Russia			1.26x10^6	
2001149	243562	42	male	Russia			positive	16.8
2001167	248173	71	female	Russia				22.2
2001178	248089	45	male	Ukraine			positive	
2001190	248835	40	male	Ukraine				17.17
2001210	246155	74	female	Russia			positive	24.7
2001212	248907	34	male	Ukraine	39 IU/ml		5.3x10^6	19
2001222	248970	61	male	Romania			9.11x10^6	18.2
2001228	248980	34	male	no info			7.52x10^7	14.7
2001234	249018	33	female	no info			1.66x10^5	24.7

S1 Table
