# Supplementary material for: Advanced molecular surveillance approaches for characterization of blood borne hepatitis viruses
Source: PLoS One. 2020 Jul 17;15(7):e0236046. doi: 10.1371/journal.pone.0236046 (PMC7367454; doi:10.1371/journal.pone.0236046)
Supplement: S2 Table — HCV positive library results are listed and include total reads, HCV reads, percent genome coverage, genotype classification, and HCV reads per million. (PDF) [file pone.0236046.s004.pdf]

Sample ID	Total Reads	HCV Reads	% Coverage	Classification	Reads/Million
1_HCV_Israeli_2000427	2,094,338	1,127	97.68	1B	538
2_HCV_Israeli_2000444	11,159,480	2,176	96.82	1B	195
3_HCV_Israeli_2000445	12,884,712	1,193	96.12	1B	93
4_HCV_Israeli_2000446	719,267	6,573	100	1B	9,138
5_HCV_Israeli_2000447	1,223,499	5,492	98.08	1B	4,489
6_HCV_Israeli_2000449	473,173	2,603	99.35	1A	5,501
7_HCV_Israeli_2000463	1,312,068	2,329	96.82	1B	1,775
8_HCV_Israeli_2000478	12,198,916	1,906	97.9	1B	156
9_HCV_Israeli_2000484	4,318,906	2,822	99.86	1B	653
10_HCV_Israeli_2000485	17,816,566	1,390	97.21	1B	78
11_HCV_Israeli_2000487	12,282,440	3	2.16	(-)	0
12_HCV_Israeli_2000502	10,128,136	14,234	100	1A	1,405
13_HCV_Israeli_2000512	9,511,250	11,322	99.95	1A	1,190
14_HCV_Israeli_2000513	8,315,644	21,476	97.33	1B	2,583
15_HCV_Israeli_2000516	5,696,236	1,961	96.3	1A	344
16_HCV_Israeli_2000517	4,862,078	3,667	97.9	1B	754
17_HCV_Israeli_2000545	3,089,350	7,959	99.35	1B	2,576
18_HCV_Israeli_2000554	11,274,782	24,033	100	1A	2,132
19_HCV_Israeli_2000561	11,104,238	6,920	98.13	1B	623
20_HCV_Israeli_2000572	7,043,992	42,511	99.95	1B	6,035
21_HCV_Israeli_2000573	11,277,786	18,129	99.28	1B-basal	1,607
22_HCV_Israeli_2000613	4,751,790	4,200	99.19	1B	884
23_HCV_Israeli_2000615	12,956,642	31,809	98.11	1B	2,455
24_HCV_Israeli_2000616	11,423,692	42,267	99.05	1A	3,700
25_HCV_Israeli_2000618	10,901,586	23,683	97.96	1B	2,172
26_HCV_Israeli_2000619	11,832,470	8,689	98.04	1B	734
27_HCV_Israeli_2000621	9,822,408	37,033	98.17	1B	3,770
28_HCV_Israeli_2000637	14,007,920	8,670	98.07	1B	619
29_HCV_Israeli_2000646	8,836,370	72,736	100	1B	8,231
30_HCV_Israeli_2000670	16,412,334	44,732	99.35	1B	2,726
31_HCV_Israeli_2000672	12,368,504	40,003	98.13	1B	3,234
32_HCV_Israeli_2000684	7,728,012	6,132	100	1B	793
33_HCV_Israeli_2000691	7,220,362	37,566	100	1B	5,203
34_HCV_Israeli_2000692	8,707,132	16,337	99.99	1B	1,876
35_HCV_Israeli_2000693	14,367,392	54,143	98.12	1B	3,768
36_HCV_Israeli_2000697	5,152,816	17,061	99.27	2C	3,311
37_HCV_Israeli_2000726	9,184,498	90,924	100	1B	9,900
38_HCV_Israeli_2000734	14,669,110	11,466	98.08	1B	782
39_HCV_Israeli_2000775	5,832,020	178,799	99.83	1B	30,658
40_HCV_Israeli_2000781	31,458,172	5	3.14	(-)	0
41_HCV_Israeli_2000782	25,823,076	12,445	99.32	1B	482
42_HCV_Israeli_2000785	33,528	65	28.14	(-)	1,939
43_HCV_Israeli_2000790	24,055,978	58,740	97.57	1B	2,442
44_HCV_Israeli_2000791	12,524,798	92,788	100	1B	7,408
45_HCV_Israeli_2000798	28,443,036	82,188	100	1B	2,890
46_HCV_Israeli_2000810	15,780,030	8,912	97.83	1A	565
47_HCV_Israeli_2000811	7,870,168	115,775	98.2	1A	14,711
48_HCV_Israeli_2000912	19,911,460	26,981	98.64	3A	1,355
49_HCV_Israeli_2000976	16,383,008	109,738	99.75	3A	6,698

S2 Table

Sample ID	Total Reads	HCV Reads	% Coverage	Classification	Reads/Million
50_HCV_Israeli_2000982	22,078,886	23,533	97.72	1B	1,066
51_HCV_Israeli_2000984	15,351,242	245,729	100	1B	16,007
52_HCV_Israeli_2000986	7,283,694	428,233	98.26	1B	58,793
53_HCV_Israeli_2001001	21,011,786	15,619	97.97	1B	743
54_HCV_Israeli_2001005	9,154,770	148,098	100	1B	16,177
55_HCV_Israeli_2001052	10,202,678	218,194	100	1B	21,386
56_HCV_Israeli_2001065	11,435,392	280,302	99.62	3A	24,512
57_HCV_Israeli_2001069	20,714,334	60,421	98.13	1B	2,917
58_HCV_Israeli_2001072	7,363,986	908,612	98.25	1B	123,386
59_HCV_Israeli_2001079	13,590,740	142,352	100	1B	10,474
60_HCV_Israeli_2001108	14,438,324	112,942	99.99	1B	7,822
61_HCV_Israeli_2001114	17,821,262	19,490	100	1B	1,094
62_HCV_Israeli_2001121	29,412,046	4,595	97.11	1B	156
63_HCV_Israeli_2001123	15,823,778	20,029	97.97	1B	1,266
64_HCV_Israeli_2001129	14,919,690	55,779	100	1B	3,739
65_HCV_Israeli_2001133	10,194,678	167,486	99.99	3A	16,429
66_HCV_Israeli_2001144	7,901,544	74,205	98.23	1B	9,391
67_HCV_Israeli_2001160	10,010,772	31,526	100	1B	3,149
68_HCV_Israeli_2001172	16,635,230	27,815	100	1A	1,672
69_HCV_Israeli_2001206	6,883,796	60,907	99.99	1A	8,848
70_HCV_Israeli_2001220	7,995,474	179,016	99.71	3A	22,390
71_HCV_Israeli_2000599	3,719,732	998	92.98%	1A	268
72_HCV_Israeli_2000602	3,416,754	1,437	98.04%	1B	421
73_HCV_Israeli_2000607	1,007,238	932	92.33%	1A	925
74_HCV_Israeli_2000633	3,785,392	4,987	99.38%	1B	1,317
75_HCV_Israeli_2000650	202,334	96	15.99%	1B	474
76_HCV_Israeli_2000668	1,186,310	13,308	98.00%	1B	11,218
77_HCV_Israeli_2000669	3,489,148	999	92.93%	1B	286
78_HCV_Israeli_2000675	4,770,178	173	43.61%	1B	36
79_HCV_Israeli_2000676	3,271,126	335	65.57%	1B	102
80_HCV_Israeli_2000694	2,821,612	28,047	99.44%	1B	9,940
81_HCV_Israeli_2000705	4,033,674	2,473	98.80%	1B	613
82_HCV_Israeli_2000883	2,170,316	4,973	97.68%	1A	2,291
83_HCV_Israeli_2000887	7,713,096	702	62.29%	1B	91
84_HCV_Israeli_2000889	4,786,486	612	79.96%	1B	128
85_HCV_Israeli_2000913	3,699,324	2,133	97.61%	1A	577
86_HCV_Israeli_2000929	2,981,288	3,365	97.44%	1A	1,129
87_HCV_Israeli_2000973	1,013,476	4,074	98.90%	1B	4,020
88_HCV_Israeli_2000989	7,815,930	8,953	98.45%	1B	1,145
89_HCV_Israeli_2001011	913,152	1,337	95.74%	1A	1,464
90_HCV_Israeli_2001044	7,862,446	2,979	86.27%	1A	379
91_HCV_Israeli_2001049	8,528,684	26,192	100.00%	3A	3,071
92_HCV_Israeli_2001067	10,999,572	2,637	95.34%	1B	240
93_HCV_Israeli_2001070	1,356,688	5,235	100.00%	1A	3,859
94_HCV_Israeli_2001088	972,990	1,892	98.41%	1B	1,945
95_HCV_Israeli_2001092	3,453,448	4,206	99.05%	3A	1,218
96_HCV_Israeli_2001106	2,892,372	17,230	99.94%	3A	5,957
97_HCV_Israeli_2001137	2,897,304	2,527	97.32%	1A	872
98_HCV_Israeli_2001159	1,270,000	505	75.44%	1B	398
99_HCV_Israeli_2001218	3,404,192	2,656	99.51%	1B	780
