# Supplementary material for: Advanced molecular surveillance approaches for characterization of blood borne hepatitis viruses
Source: PLoS One. 2020 Jul 17;15(7):e0236046. doi: 10.1371/journal.pone.0236046 (PMC7367454; doi:10.1371/journal.pone.0236046)
Supplement: S3 Table — Pairwise nucleotide identity values comparing HDV consensus sequences from mNGS versus xGen is expressed as a percent. (PDF) [file pone.0236046.s005.pdf]

Pairwise nucleotide identity between HDV mNGS and xGen consensus sequences

Specimen ID	% identity	gapstripped alignment
2000166	87.02532	314
2000208	99.34598	1527
2000234	96.30819	623
2000236	98.63192	1532
2000319	91.71843	965
2000320	99.47917	192
2000322	97.34848	1578
2000324	96.61164	1150
2000372	99.46381	1492
2000563	98.84511	1470
2000570	99.33378	1501
2000741	91.01449	345
2000742	96.30404	1431
2000744	99.61165	1545
2000999	99.81774	1646
2001063	99.47678	1528
2001073	99.42122	1554
2001149	99.44751	1628
2001167	99.0572	1591
2001178	99.60707	1526
2001190	99.75728	1648
2001210	99.67448	1535
2001212	96.46279	1353
2001222	98.1685	1364
2001228	98.92857	1679
2001234	98.52399	1355
