# Supplementary material for: Advanced molecular surveillance approaches for characterization of blood borne hepatitis viruses
Source: PLoS One. 2020 Jul 17;15(7):e0236046. doi: 10.1371/journal.pone.0236046 (PMC7367454; doi:10.1371/journal.pone.0236046)
Supplement: Attachment — Submitted filename: Response to Reviewers.docx [file pone.0236046.s006.docx]

PONE-D-20-06995
Advanced Molecular Surveillance Approaches for Characterization of Blood Borne Hepatitis Viruses
PLOS ONE

Dear Dr. Yury E Khudyakov PhD,

Thank you kindly for the very fruitful review process and for the needed extension to make revisions. I believe we have addressed all the journal’s and reviewers’ concerns. Figure 3 has been modified and we’ve added a supplemental table (S1) with demographic data. Figure S2 has been removed and previous supplemental tables have been renumbered. Finally, sequences have been deposited in GenBank and accession numbers are noted. We look forward to hearing back from you that the manuscript is now acceptable.

Kind regards,

Michael Berg, PhD

Abbott Laboratories

*********************************************************************************

Journal Requirements:

When submitting your revision, we need you to address these additional requirements.

1. Please ensure that your manuscript meets PLOS ONE's style requirements, including those for file naming. The PLOS ONE style templates can be found at

<https://journals.plos.org/plosone/s/file?id=wjVg/PLOSOne_formatting_sample_main_body.pdf> and

<https://journals.plos.org/plosone/s/file?id=ba62/PLOSOne_formatting_sample_title_authors_affiliations.pdf>

2. Please specify whether you obtained IRB approval for use of specimens collected from volunteer blood donors in Spain and Cameroon, and if so, the full name of the IRB that approved this.

Spanish samples were selected from the Biobank of the Catalonia Blood Bank and de-identified. IRB approval was obtained from the Vall d’Hebron Hospital Ethics Committee.

Cameroonian samples were from two HBV surveillance studies conducted from 2010–2016. These were approved by the Ministry of Health of Cameroon, the Cameroon National Ethical Review Board, and the Faculty of Medicine and [Biomedical Science](https://www.sciencedirect.com/topics/medicine-and-dentistry/biomedical-discipline) IRB.

3. Please provide additional details regarding participant consent in the ethics statement in the Methods.

Please ensure that you have specified (i) whether consent was informed and (ii) what type you obtained (for instance, written or verbal, and if verbal, how it was documented and witnessed).

If your study included minors, state whether you obtained consent from parents or guardians.

If the need for consent was waived by the ethics committee, please include this information.

For Israel, patient plasma was collected from individuals seeking treatment at the Israeli National HIV and Viral Hepatitis Reference Center (NHRL). Included were the remains of plasma samples from patients that were referred to the laboratory for HDV viral load measurements (HBV-HDV co-infections) or for HCV RAS analysis. These were exempt from signing a consent form by the local IRB. The IRB approval numbers for HCV and for HDV are 9329-12-SMC and 2890-15-SMC, respectively)

For Spain, participants were recruited from Barcelona and provided written, informed consent. For Cameroon, participants were recruited from blood bank donors, hospitals, and chest clinics in the urban centers of Douala and Yaoundé. Written informed consent was provided and plasma was collected anonymously.

4. In your Methods section, please provide additional information about the participant recruitment method and the demographic details of your participants. Please ensure you have provided sufficient details to replicate the analyses such as:

a) the recruitment date range (month and year), HDV samples were collected from October 2014- Jul 2017. HCV samples were collected from June 2015-Jul 2017.

b) a description of any inclusion/exclusion criteria that were applied to participant recruitment, Inclusion criteria (only): DAA naïve HCV-RNA positive patients and HBV+ HDV detectable HDV RNA

c) a table of relevant demographic details, Demographic details are now included in a separate Table in the online supplement. This information is also present in each GenBank entry.

d) a statement as to whether your sample can be considered representative of a larger population, and

These samples were randomly collected from patients during the time period mentioned in 4a and can be considered representative of a larger HCV positive or HBV/HDV dually positive population.

e) a description of how participants were recruited.

Individuals seeking treatment at the Israeli National HIV and Viral Hepatitis Reference Center (NHRL) and meeting the inclusion criteria in 4b were recruited to the study.

5. We note that you have included the phrase “data not shown” in your manuscript. Unfortunately, this does not meet our data sharing requirements. PLOS does not permit references to inaccessible data. We require that authors provide all relevant data within the paper, Supporting Information files, or in an acceptable, public repository. Please add a citation to support this phrase or upload the data that corresponds with these findings to a stable repository (such as Figshare or Dryad) and provide and URLs, DOIs, or accession numbers that may be used to access these data. Or, if the data are not a core part of the research being presented in your study, we ask that you remove the phrase that refers to these data.

 This phrase was removed.

6. We note that you have stated that you will provide repository information for your data at acceptance. Should your manuscript be accepted for publication, we will hold it until you provide the relevant accession numbers or DOIs necessary to access your data. If you wish to make changes to your Data Availability statement, please describe these changes in your cover letter and we will update your Data Availability statement to reflect the information you provide.

Accession numbers for HCV sequences are MT632105-MT632194.

Accession numbers for HBV sequences are MT622522-MT622525.

Accession numbers for HDV sequences are MT583788-MT583813.

7. Thank you for stating the following in the Financial Disclosure section:

'The authors were funded by Abbott Laboratories.'

We note that one or more of the authors have an affiliation to the commercial funders of this research study, Abbott Laboratories.

a. Please provide an amended Funding Statement declaring this commercial affiliation, as well as a statement regarding the Role of Funders in your study. If the funding organization did not play a role in the study design, data collection and analysis, decision to publish, or preparation of the manuscript and only provided financial support in the form of authors' salaries and/or research materials, please review your statements relating to the author contributions, and ensure you have specifically and accurately indicated the role(s) that these authors had in your study. You can update author roles in the Author Contributions section of the online submission form.

Please also include the following statement within your amended Funding Statement.

“The funder provided support in the form of salaries for authors [insert relevant initials], but did not have any additional role in the study design, data collection and analysis, decision to publish, or preparation of the manuscript. The specific roles of these authors are articulated in the ‘author contributions’ section.”

This new language has been added to our amended Funding Statement.

If your commercial affiliation did play a role in your study, please state and explain this role within your updated Funding Statement.

 Our commercial affiliation did not play a role in the study.

b. Please also provide an updated Competing Interests Statement declaring this commercial affiliation along with any other relevant declarations relating to employment, consultancy, patents, products in development, or marketed products, etc.

Within your Competing Interests Statement, please confirm that this commercial affiliation does not alter your adherence to all PLOS ONE policies on sharing data and materials by including the following statement: "This does not alter our adherence to  PLOS ONE policies on sharing data and materials.” (as detailed online in our guide for authors [http://journals.plos.org/plosone/s/competing-interests](about:blank)). If this adherence statement is not accurate and  there are restrictions on sharing of data and/or materials, please state these. Please note that we cannot proceed with consideration of your article until this information has been declared.

We declare in the Competing Interests Statement regarding our commercial affiliation: "This does not alter our adherence to PLOS ONE policies on sharing data and materials.” (as detailed online in our guide for authors [http://journals.plos.org/plosone/s/competing-interests](about:blank)).

c. Please include both an updated Funding Statement and Competing Interests Statement in your cover letter. We will change the online submission form on your behalf.

An updated Funding Statement and Competing Interests Statement has been added to our cover letter.

Please know it is PLOS ONE policy for corresponding authors to declare, on behalf of all authors, all potential competing interests for the purposes of transparency. PLOS defines a competing interest as anything that interferes with, or could reasonably be perceived as interfering with, the full and objective presentation, peer review, editorial decision-making, or publication of research or non-research articles submitted to one of the journals. Competing interests can be financial or non-financial, professional, or personal. Competing interests can arise in relationship to an organization or another person. Please follow this link to our website for more details on competing interests: [http://journals.plos.org/plosone/s/competing-interests](about:blank)

[Note: HTML markup is below. Please do not edit.]

Reviewers' comments:

Reviewer's Responses to Questions

**Comments to the Author**

1. Is the manuscript technically sound, and do the data support the conclusions?

The manuscript must describe a technically sound piece of scientific research with data that supports the conclusions. Experiments must have been conducted rigorously, with appropriate controls, replication, and sample sizes. The conclusions must be drawn appropriately based on the data presented.

Reviewer #1: Yes

Reviewer #2: Yes

2. Has the statistical analysis been performed appropriately and rigorously?

Reviewer #1: N/A

Reviewer #2: N/A

3. Have the authors made all data underlying the findings in their manuscript fully available?

The [PLOS Data policy](http://www.plosone.org/static/policies.action#sharing) requires authors to make all data underlying the findings described in their manuscript fully available without restriction, with rare exception (please refer to the Data Availability Statement in the manuscript PDF file). The data should be provided as part of the manuscript or its supporting information, or deposited to a public repository. For example, in addition to summary statistics, the data points behind means, medians and variance measures should be available. If there are restrictions on publicly sharing data—e.g. participant privacy or use of data from a third party—those must be specified.

Reviewer #1: Yes

Reviewer #2: Yes

4. Is the manuscript presented in an intelligible fashion and written in standard English?

PLOS ONE does not copyedit accepted manuscripts, so the language in submitted articles must be clear, correct, and unambiguous. Any typographical or grammatical errors should be corrected at revision, so please note any specific errors here.

Reviewer #1: Yes

Reviewer #2: Yes

5. Review Comments to the Author

Please use the space provided to explain your answers to the questions above. You may also include additional comments for the author, including concerns about dual publication, research ethics, or publication ethics. (Please upload your review as an attachment if it exceeds 20,000 characters)

Reviewer #1: The present manuscript reports on HCV, HBV, and HDV molecular epidemiology in infected patients from Israel by using the NGS MiSeq system to obtain full genome sequences. HCV sequencing was successfully performed directly on 99 samples carrying relatively high viral RNA loads as expected. Phylogenetic analysis indicated that genotype 1 was dominant in these patients (clinical status?) with subgenotype differences between patients born in Israel (HCV-1a) and patients originating from Russia and Eastern Europe. Diversity was illustrated further by the presence of genotypes 2-4. It would be interesting to know if there was difference in genome coverage and HCV reads/million according to genotypes.

The answer can be found in **Supplemental Table S1**. All the 2c and 3a sequences had >98% genome coverage and >1200 rpm. A few had <97% genome coverage, and these were genotype 1A or 1B, which made up the majority of samples.

It is unexpected that the authors did not report the detection of any pegivirus which are quite prevalent in humans.

We regret the confusion here; an initial version of the text attempted to make the following distinction, which we ought to have kept in. HPgV-**2** is a novel pegivirus that our lab discovered in 2015: it is primarily seen in HCV infections, so we were looking specifically for this virus, but we did not detect it in these HCV+ individuals. We did however observe human pegivirus-**1**, also known as **GBV-C**, in 5 individuals. As the reviewer notes, it is quite common. We have included the following sentence on lines 264-265, “Human pegivirus-1 (GBV-C) reads were enriched in 5 individuals.” We also further clarify this in the Discussion on lines 389-91,

“Sensitivity for numerous viruses in our controls suggested we should have detected other blood borne pathogens if they were there (S1 Fig), but no additional co-infections **besides GBV-C** were present among the HCV positive individuals.”

In parallel, 26 samples previously documented HBV/HDV co-infected samples were investigated. Due to the extremely low level of HBV DNA in these samples, two methods of viral targets enrichment were evaluated. xGen probe sets (2x coverage) for the main HBV and HDV genotypes were designed and combined in the same assay. The method was first evaluated on a panel of characterized HBV mono-infected and HBV/HDV dual-infected samples. The results obtained with both panel and patient samples showed the efficiency of the method to recover HBV sequences. The use of a commercial pan viral probe set for HBV was less efficient showing the importance to use probes covering the genetic diversity of all HBV genotypes. Target enrichment showed poor added value for HDV sequencing. The HBV and HDV genotypes observed were consistent with previous data from the region. An interesting feature is the identification of a rare HBV-BD recombinant strain. However, the Simplot profile was derived from a consensus sequence generated by NGS. How the authors excluded the possibility of dual infection? Did they check that there was no overlapping reads characteristic of the two genotypes?

We thank the reviewer for their very astute inquiry. After careful scrutiny of the data, we have reached the conclusion that indeed it is not a recombinant, but rather a dual infection. As they noted, there were overlapping reads indicative of both genotypes, but because a consensus sequence was used for phylogenetics and SIMPLOT analysis, these less abundant reads were obscured by the majority sequence. As the plot in Fig 3A indicated and we noted in the text, coverage was incomplete at 69%. Upon closer inspections, the transition from one genotype to the other occurred at these gaps. In addition, there were no contiguous reads that spanned the putative ‘breakpoints’. Rather, one could see where reads from one genotype ended and a new set of reads for the other genotype began. We regret this oversight and are grateful to the reviewer for raising this possibility and allowing us to catch this mistake.

We have modified the abstract (lines 51-54) to read as follows:

When HBV-xGen was applied to Israeli samples, coverage was improved by 28-73% in 4 samples and identified HBV genotype A1, A2, D1 specimens and a ~~previously never described BD recombinant~~ dual B/D infection.

We have modified the results (lines 339-343):

Before

Interestingly, this strain was classified as a rare BD recombinant, which we confirmed by bootscanning in SIMPLOT and individual trees of fragments (Fig 3B; S2 Fig). A separate bleed from this individual did not yield HBV sequence, but prior PCR confirmed D1 sequence in the RT region.

After

Interestingly, this individual was dually-infected with genotypes B2 and D1. Incomplete coverage and reliance on a consensus sequence created the appearance of a recombinant, however, overlapping sequences from both strains were detected whereas contiguous reads spanning putative ‘recombination breakpoints’ were not. (Fig 3A).

Note that the original Fig 3B has been removed, as well the accompanying phylogenetic trees of sub-fragments in Fig S2. In its place, Fig 3A now includes a lower panel that illustrates which HBV genotype reads are coming from.

The legend for Fig 3A now reads as follows:

(A) Coverage plot of HBV specimen, 2001149. mNGS reads are shown in orange and xGen reads in dark blue (upper panel). Genotype D1 reads are in gold and genotype B2 reads are in brown (lower panel).

We have modified the Discussion now to remove two sentences (lines 437-441) and it reads as follows:

Where HBV sequences were obtained, these were genotype A1, A2, D, and a ~~BD recombinant~~ B/D dual infection. ~~Recombinants of HBV are themselves rare, and a BD mosaic genome has never been described until here (Fig 3B, S2 Fig) [39]. Consistent with reports that recombination sites are not distributed randomly in HBV, we observed breakpoints within in the 1700-2000 region.~~

Reviewer #2: In this manuscript, the authors presented a metagenomic next generation sequencing approach (mNGS) for the molecular characterization of blood borne viral hepatitis viruses (HCV, HBV and HDV).
Overall, the methods employed by the authors to accomplish their objectives were adequate and well-presented. The viral enrichment method, crucial for mNGS and a topic of great interest for virological studies, presented solid results when applied in the international HBV panel but not so consistent regarding Israeli samples. Nonetheless, the technique allowed to detect a previously unreported recombinant B/D strain which was a great achievement. The tables presented in the article should be clearer. It lacks proper legend to understand what hyphen means in many lines (not done, not available?).
The employment of new methodologies to provide tools for virological surveillance is relevant for scientific community and this paper surely contributes to a remarkable bottleneck of viral metagenomics which is the low sensibility of detection of low-titer viral DNA/RNA. However, as highlighted by the authors, its use in routine diagnosis does not appear imminent, especially in low income countries.
The quality of the written English is good thorough out the article.


Minor revision

1. Table 1 is not self-explanatory and should be clearer. Only in the text it is possible to understand that the values shown are either absolute numbers or percentages. This must be clearly indicated in the table.

The columns headers in Table 1 have been modified to indicate the absolute number of total individuals.

2. The description of the cohort in line 381 repeats the male gender twice. Authors must correct the typing error.

It now reads “…53 males and 43 females…”

3. A typo in the word “Kazakhstan” (line 391) must be corrected.

Corrected.

6. PLOS authors have the option to publish the peer review history of their article ([what does this mean?](https://journals.plos.org/plosone/s/editorial-and-peer-review-process#loc-peer-review-history)). If published, this will include your full peer review and any attached files.


If you choose “no”, your identity will remain anonymous but your review may still be made public.


**Do you want your identity to be public for this peer review?** For information about this choice, including consent withdrawal, please see our [Privacy Policy](https://www.plos.org/privacy-policy).

Reviewer #1: No

Reviewer #2: No


[NOTE: If reviewer comments were submitted as an attachment file, they will be attached to this email and accessible via the submission site. Please log into your account, locate the manuscript record, and check for the action link "View Attachments". If this link does not appear, there are no attachment files to be viewed.]


While revising your submission, please upload your figure files to the Preflight Analysis and Conversion Engine (PACE) digital diagnostic tool, <https://pacev2.apexcovantage.com/>. PACE helps ensure that figures meet PLOS requirements. To use PACE, you must first register as a user. Registration is free. Then, login and navigate to the UPLOAD tab, where you will find detailed instructions on how to use the tool. If you encounter any issues or have any questions when using PACE, please email us at [figures@plos.org](mailto:figures@plos.org). Please note that Supporting Information files do not need this step.
